# Bacteria and phage consortia modulate cecal SCFA production and host metabolism to enhance feed efficiency in ducks

**DOI:** 10.1186/s40168-026-02368-y

**Published:** 2026-03-04

**Authors:** Xia Wang, Shujie Tian, Yunsheng Zhang, Li Yang, Di Hu, Zezhong Wang, Xueqin Yang, Shaofei Li, Jie Wei, Wei Zhou, Shuaiqin Wang, Li Deng, Fuyong Li, Shuisheng Hou, Pengying Li, Jinlong Ru

**Affiliations:** 1 College of Animal Science and Technology, Northwest A&F University, Yangling, 712100 Shaanxi China; 2 Institute of Animal Sciences, Chinese Academy of Agricultural Sciences, Beijing, 100193 China; 3 Prevention of Microbial Diseases, TUM School of Life Sciences; Central Institute of Infection Prevention (ZIP), Technical University of Munich, Freising, 85354 Germany; 4 Research Unit of Microbial Diseases Prevention, Institute of Virology, Helmholtz Centre Munich-German Research Centre for Environmental Health, Neuherberg, 85764 Germany; 5 Department of Animal Science and Technology, College of Animal Sciences, Zhejiang University, Hangzhou, 310058 China; 6 Ministry of Education Key Laboratory of Molecular Animal Nutrition, Zhejiang University, Hangzhou, 310058 China; 7 College of Landscape Architecture and Art, Northwest A&F University, Yangling, 712100 China

## Abstract

**Background:**

The gut microbiota influences poultry health, nutrition, feed efficiency (FE), and overall productivity. However, the relationship between gut microbes, including bacteria and phages, and FE in ducks remains underexplored. To address this, we integrated cecal 16S amplicon, metagenome, microbiota-derived short-chain fatty acids (SCFAs) profiling, liver transcriptome, and serum metabolome data to illustrate the contribution of the gut microbiome (bacteria and viruses) to duck FE.

**Results:**

We reconstructed viral genomes and prokaryotic metagenome-assembled genomes (MAGs) and annotated their genes using comprehensive databases. Prokaryotic hosts of viruses were also predicted to understand virus-host dynamics within the gut ecosystem. Our results revealed that high-FE ducks have higher concentration of propionate and butyrate in cecum compared with low-FE ducks. The metagenome sequencing revealed distinct cecal microbiota profiles between two groups, with increased relative abundance of representative SCFA producers, especially *Paraprevotella sp905215575* and *Bacteroides sp944322345*, and enhanced SCFA-biosynthesis pathways in high-FE ducks. Virome genome assembly identified two phages encoding auxiliary metabolic genes (AMGs) involved in pyruvate metabolism, enhancing nutrient availability for host bacteria to produce SCFAs (e.g., temperate phage-encoded pyruvate phosphate dikinase) or exploiting host central metabolic pathways for viral replication (e.g., lytic phage-encoded formate C-acetyltransferase). Furthermore, these representative SCFA-producing bacteria and phage consortia were associated with serum metabolites (including L-histidine and 4-hydroxydecanedioylcarnitine) linked to duck FE.

**Conclusion:**

Collectively, these findings provide novel insights into the gut microbial factors regulating FE in ducks, offering potential strategies to optimize poultry nutrition and productivity.

Video Abstract

**Supplementary Information:**

The online version contains supplementary material available at 10.1186/s40168-026-02368-y.

## Introduction

Efficient feed utilization is critical for sustainable and profitable poultry production. Feed costs account for 60–70% of total production expenses in commercial poultry operations, making feed efficiency (FE) a primary determinant of economic viability [1]. In the context of a growing global demand for animal protein, enhancing FE in poultry species like chickens and ducks holds significant economic and environmental importance through lower feed waste and nitrogen excretion.

In non-ruminant animals like poultry and pigs, short-chain fatty acids (SCFAs), primarily produced through microbial fermentation of fiber in the cecum or colon, can supply 60–70% of the energy requirements of intestinal epithelial cells [2]. This process not only sustains normal intestinal epithelial function but also plays a vital role in overall gut health and metabolic regulation. Beyond energy provision, SCFAs (such as acetate, propionate, and butyrate) function as signaling molecules by activating G protein-coupled receptors (GPCRs), including FFAR2 (GPR43), FFAR3 (GPR41), and HCAR2 (GPR109A). Activation of these receptors modulates energy expenditure, preadipocyte differentiation, and appetite control [3, 4]. Given these functions, modulating the gut microbiota to enhance SCFA production represents a promising strategy for improving feed utilization. For example, probiotic supplementation promotes a faster shift toward a mature, stable microbiota composition, increasing average daily gain (ADG) by 6.2% while reducing FCR by 4.5% compared to antibiotic-free controls [5]. Similarly, microbiota-accessible fibers boost populations of SCFA-producing bacteria (e.g., *Bacteroidetes*) by up to 40%, enhancing energy harvest and intestinal barrier function in laying hens [6]. While bacterial contributions to FE through SCFA production are well-documented [7–9], the specific roles of phages in modulating these bacterial functions and their combined impact on duck feed efficiency remain uncharacterized, preventing the development of comprehensive microbiome-based strategies to optimize feed utilization.

The gut virome, dominated by phages (viruses infecting bacteria), represents one of the densest viral ecosystems in nature, with estimates of more than 10^8^ to 10^10^ virus-like particles per gram of intestinal content [10, 11]. These phages exert profound influence over microbial community dynamics and metabolic processes through predator–prey interactions, horizontal gene transfer, and the regulation of bacterial lysogenic-to-lytic life cycles. For instance, phages can reshape bacterial diversity by selectively lysing dominant taxa like *Bacteroides* or *Bacillota*, thereby altering niche competition and nutrient flow [12]. Phage-encoded auxiliary metabolic genes (AMGs) further modulate bacterial metabolism, such as enhancing carbohydrate utilization in *Bacteroides* [13] and *Oscillospiraceae* [14]. Such phage activities can cascade to reshape microbial functional outputs, such as bile acid metabolism and SCFA production, with indirect implications for host physiology.

Despite their ubiquity and functional significance, the role of gut viruses in shaping host phenotypes remains understudied outside human disease contexts (e.g., inflammatory bowel disease, where phage dysbiosis alters bacterial consortia and immunity [15]). In livestock, emerging evidence suggests agricultural relevance: rumen phages in cattle may regulate host nutrient utilization via AMGs and virus-host linkages, potentially influencing FE [12, 16]. These findings position the virome as a hidden effector bridging microbial ecology and host biology. However, data on viral-bacterial interactions and their association with FE in ducks are limited, and specific microbial taxa or functional genes linked to duck FE remain unidentified. Elucidating these interactions is essential for developing microbiome-based strategies to enhance FE in ducks.

This study characterized gut microbial communities, including bacteria and phages, in ducks stratified by FE. We hypothesized that high-FE ducks harbor distinct gut microbiota compositions and functional capabilities compared with low-FE ducks. We further aimed to resolve virus-host dynamics and assess their impact on bacterial community structure and function. By integrating 16S amplicon, metagenome, SCFAs, liver transcriptome, and serum metabolome data, we provide a comprehensive assessment of the duck gut microbiome’s association with FE. Our findings may inform microbiome-targeted strategies (e.g., probiotics, dietary interventions) to optimize feed utilization in duck production.

## Results

### FE traits and SCFAs in high-FE (ZF) and low-FE (CF) ducks

**Fig. 1:**
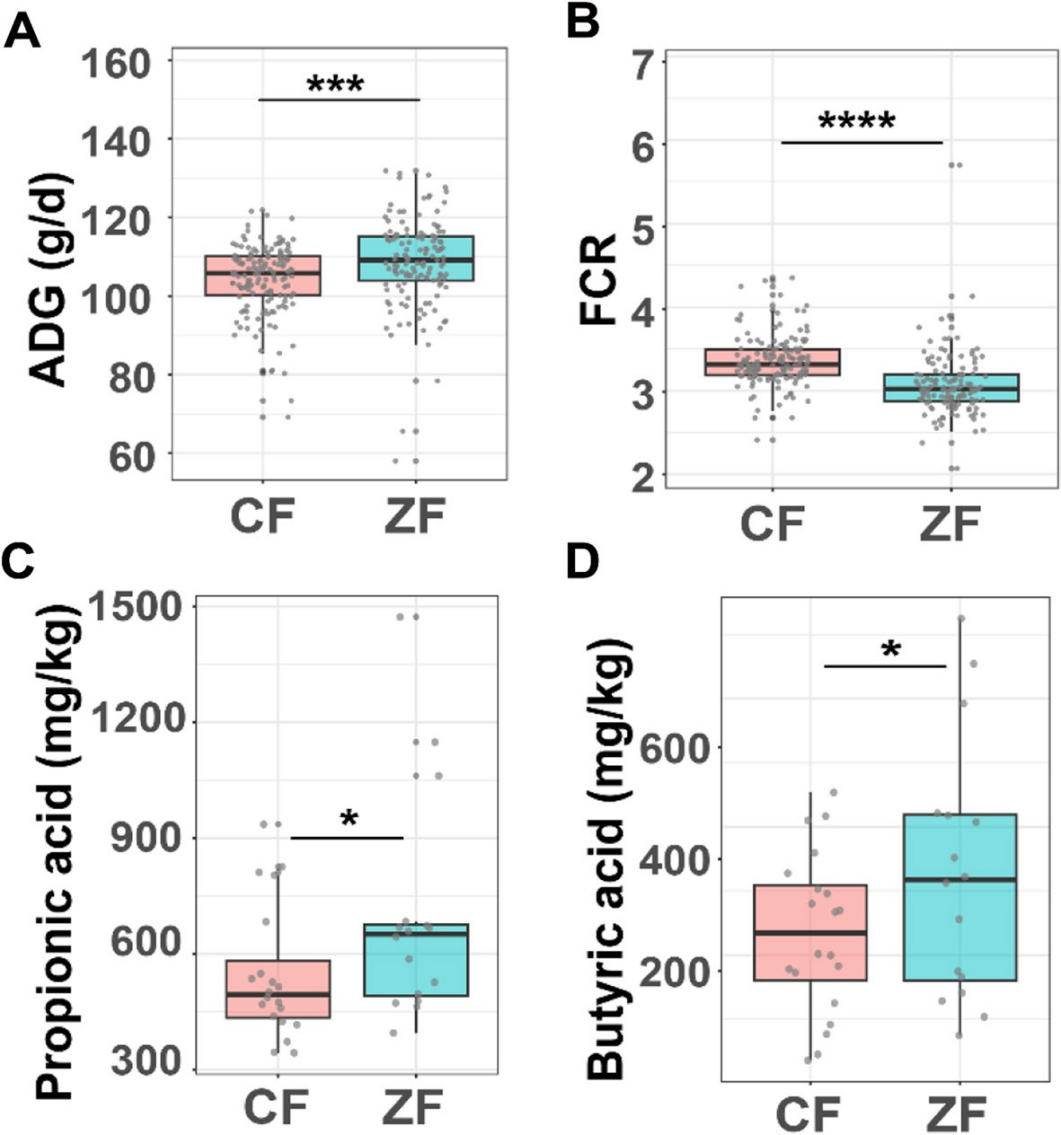
FE traits and SCFAs in two groups. **A** and **B** Average daily gain (ADG) and feed conversion ratio (FCR) of CF and ZF ducks. **C** and **D** Absolute concentrations of propionate and butyrate in two groups. *p < 0.05, ***p < 0.001, ****p < 0.0001

Two Pekin duck populations with divergent FE were used: a high-FE group (ZF, *n* = 150) and a low-FE group (CF, *n* = 150) (see the “Methods” section for details). The statistics of average daily feed intake (ADFI), average daily gain (ADG), feed conversion ratio (FCR), and fatness traits between the two duck populations are shown in Fig. 1A, B and Table S1. Compared to ZF ducks, CF ducks exhibited 5.45% higher ADFI (*p* < 0.0001), 4.59% lower ADG (*p* < 0.001), 9.45% higher FCR (*p* < 0.0001), 55.83% greater skin fat weight (SFW) (*p* < 0.001), and 26.64% greater abdominal fat weight (AFW) (*p* < 0.001). These results demonstrate significantly superior FE and reduced adiposity in the ZF population relative to CF ducks.

SCFAs derived from gut microbiota have been proven to be associated with animal FE previously [8, 9]. To compare cecal SCFAs in two groups, we analyzed 10 SCFAs from a randomly selected subset of both groups (20 CF, 16 ZF), which also showed a significant difference in FCR and ADG (Fig. S1A, B), using targeted metabolomics. We found that ZF ducks exhibited higher propionate and butyrate concentrations in cecal content compared with CF ducks (*p* < 0.05), while the others had no significant difference (Fig. 1C, D, and Fig. S1C–J). These results combined suggest that higher propionate and butyrate are associated with improved FE in ducks.

### Divergence of cecal bacterial diversity, composition, and function in high- and low-FE ducks

**Fig. 2:**
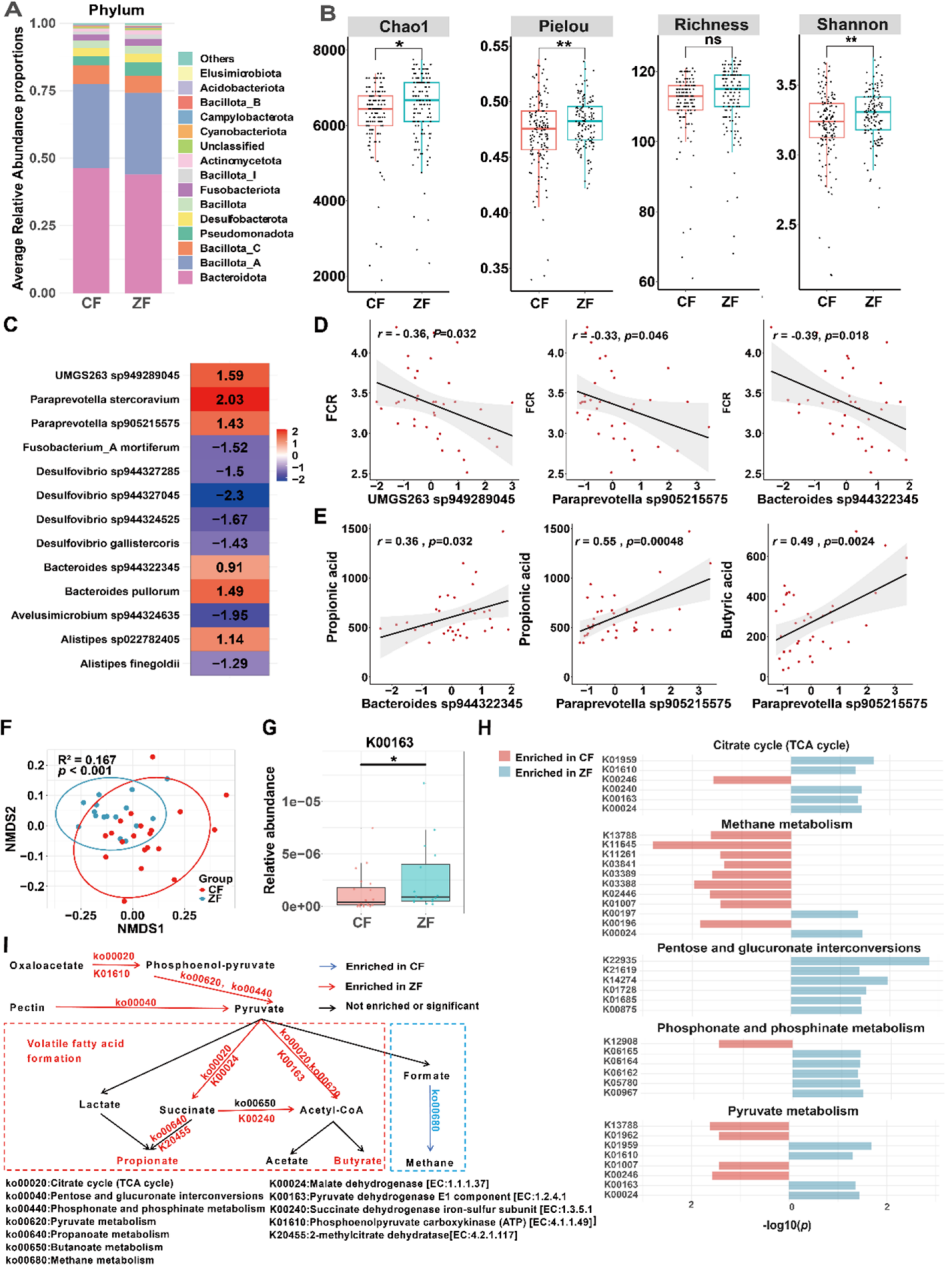
Taxonomic compositions, diversity, and co-occurrence networks of gut bacterial communities revealed by 16S rRNA gene amplicon sequencing. **A** The average composition of top 15 bacterial communities at phylum level in CF and ZF duck populations. *p < 0.05, **p < 0.01. **B** α-diversity (Chao1, Pielou, Richness, and Shannon indices) in two groups. **C** Differential abundance of bacterial species between two groups (*p* < 0.05). Values represent natural log fold changes from ANCOM-BC analysis. The color gradient indicates the direction and magnitude of change: blue to white represent decreases, while white to red represent increases in ZF compared with CF. **D** Pearson correlation and linear regression analyses between bacterial species and feed conversion ratio (FCR). **E** Correlation and linear regression analyses between bacterial species and SCFAs (butyrate and propionate). The correlation coefficient r and the corresponding *p*-value are displayed in each plot. The regression line (gray) is shown with its 95% confidence interval (shaded area). **F** Non-metric multidimensional scaling (NMDS) analysis of the Bray–Curtis distances based on gut microbial species. Ellipsoids represent a 95% confidence interval surrounding each group. **G** Relative abundance (total sum scaling) of aceE (K00163; pyruvate dehydrogenase) in two groups. **H** Bar plot showing the *p*-values (-log10 transformed) of differentially abundant KOs in modules. Red, ZF-enriched KOs; blue, CF-enriched KOs. **I** Metabolic pathways involved in SCFAs biosynthesis and methanogenesis

SCFAs in duck are primarily derived from microbial fermentation of fiber in the cecum; we therefore first investigated cecal bacterial composition and interactions in CF and ZF groups using 16S rRNA gene amplicon sequencing. The data revealed a conserved phylum-level structure across both groups, dominated by *Bacteroidetes* followed by *Bacillota_A* and *Bacillota_C* (Fig. 2A and Fig. S2A–F). While observed ASV richness was comparable between groups, ZF ducks exhibited significantly elevated α-diversity versus CF ducks as measured by Chao1, Pielou, and Shannon indices (Fig. 2B). The co-occurrence networks of the two groups have low adjusted rand index (ARI) (0.136) [17], indicating that the network’s structural and topological characteristics varied for each group. Detailed information on network topology is shown in Fig. S2G and Tables S2–S3.

To further identify the specific intestinal microbial species that contribute to FE, we analyzed cecal microbiome from the subset of both groups (20 CF, 16 ZF) using metagenomics. Prokaryotic genome reconstruction from metagenomic data yielded 1820 MAGs through single-sample assembly (mean contigs per MAG 482). Quality filtering (completeness ≥ 50%, contamination < 10%) retained 943 medium-to-high-quality MAGs. Taxonomic classification assigned these MAGs to 16 phyla, with 890 MAGs (94.4%) resolving to 121 genera and 620 MAGs to 144 known species. Notably, 321 MAGs (34.0%) lacked matches to reference genomes, representing potentially novel species.

We performed the analysis of the composition of microbiomes with bias correction (ANCOM-BC) and identified 13 species significantly different in terms of abundance between two groups (adjusted *p* < 0.05) (Fig. 2C). Specifically, gut bacterial communities in ZF ducks were mainly enriched in these species, such as *UMGS263 sp949289045*, *Paraprevotella stercoravium*, *Paraprevotella sp905215575*, *Bacteroides pullorum*, and *Bacteroides sp944322345*, while CF animals were more abundant in *Alistipes finegoldii*, *Desulfovibrio sp944324525*, *Fusobacterium A mortiferum*, and *Avelusimicrobium sp944324635*. We then performed correlation analyses between these differentially abundant bacterial species and FCR/ADG among individuals. Multiple species, including *UMGS263 sp949289045*, *Paraprevotella sp905215575*, and *Bacteroides sp944322345*, were negatively correlated with FCR (*r* = − 0.43 ~ − 0.37, *p* < 0.05) but positively correlated with ADG (Fig. 2D and Fig. S2H). We further analyzed the correlation of these differential bacterial species with propionate or butyrate; the results revealed multiple differential bacteria, such as *Paraprevotella sp905215575* and *Bacteroides sp944322345*, were positively correlated with propionate or butyrate (*r* = 0.36 ~ 0.55, *p* < 0.05), while a few species, including *Desulfovibrio sp944324525* and *Alistipes finegoldii*, displayed a negative association with propionate (Fig. 2E and Fig. S2H).

To further compare the functional features of microbiota in two duck groups, we annotated the microbial functions of each metagenome via the KEGG database using a gene-centric approach [18] and quantified the abundance of each KEGG orthologous (KOs) in different samples. Non-metric multidimensional scaling (NMDS) plot showed an obvious difference in the gut microbial KOs between the CF and ZF groups (PERMANOVA, *R*^2^ = 0.167, *p* < 0.001; Fig. 2F). We identified 545 differentially abundant KOs (*p* < 0.05), with 339 (62.2%) enriched in ZF ducks. These differential KOs were significantly enriched in multiple KEGG pathways including “carbon metabolism”, “sulfur metabolism”, “phosphonate and phosphinate metabolism”, “methane metabolism”, “citrate cycle (TCA cycle)”, “nucleotide metabolism”, and “pyruvate metabolism” (adjusted *p* < 0.1, Fig. S2I). As “pyruvate metabolism” is a central hub in the anaerobic glycolysis pathway of gut bacteria, driving the synthesis of short-chain fatty acids (SCFAs) like acetate, propionate, and butyrate, ZF microbiomes showed significant enrichment of aceE (K00163; pyruvate dehydrogenase) within pyruvate oxidation modules (M00307), converting pyruvate to acetyl-CoA, the central precursor for SCFA biosynthesis (Fig. 2G). This predicted enhanced SCFA production was consistent with our experimental measurement that ZF ducks exhibited higher propionate and butyrate concentrations in cecal content compared with CF ducks.

Regarding carbohydrate metabolism and energy metabolism, KEGG pathway enrichment analysis (Fig. S2I) revealed that the differential KOs were significantly enriched in several pathways. Notably, the “methane metabolism” pathway (in which 9 of the 12 differential KOs were higher in CF) was predominantly enriched in the CF microbiome. Conversely, the “Citrate cycle (TCA cycle)” (5/6 KOs enriched in ZF), “Pentose and glucuronate interconversions” (6/6 KOs enriched in ZF), and “Phosphonate and phosphinate metabolism” (5/6 KOs enriched in ZF) pathways were significantly more abundant in the gut microbiome of the ZF group (Fig. 2H, I). These enrichment patterns align with known energy-partitioning mechanisms: ZF’s enhanced pentose/glucuronate interconversions promote SCFA yield [19, 20], while CF’s methane metabolism redirects carbon from SCFA production [21].

### Gut virome characterization and host interactions

**Fig. 3:**
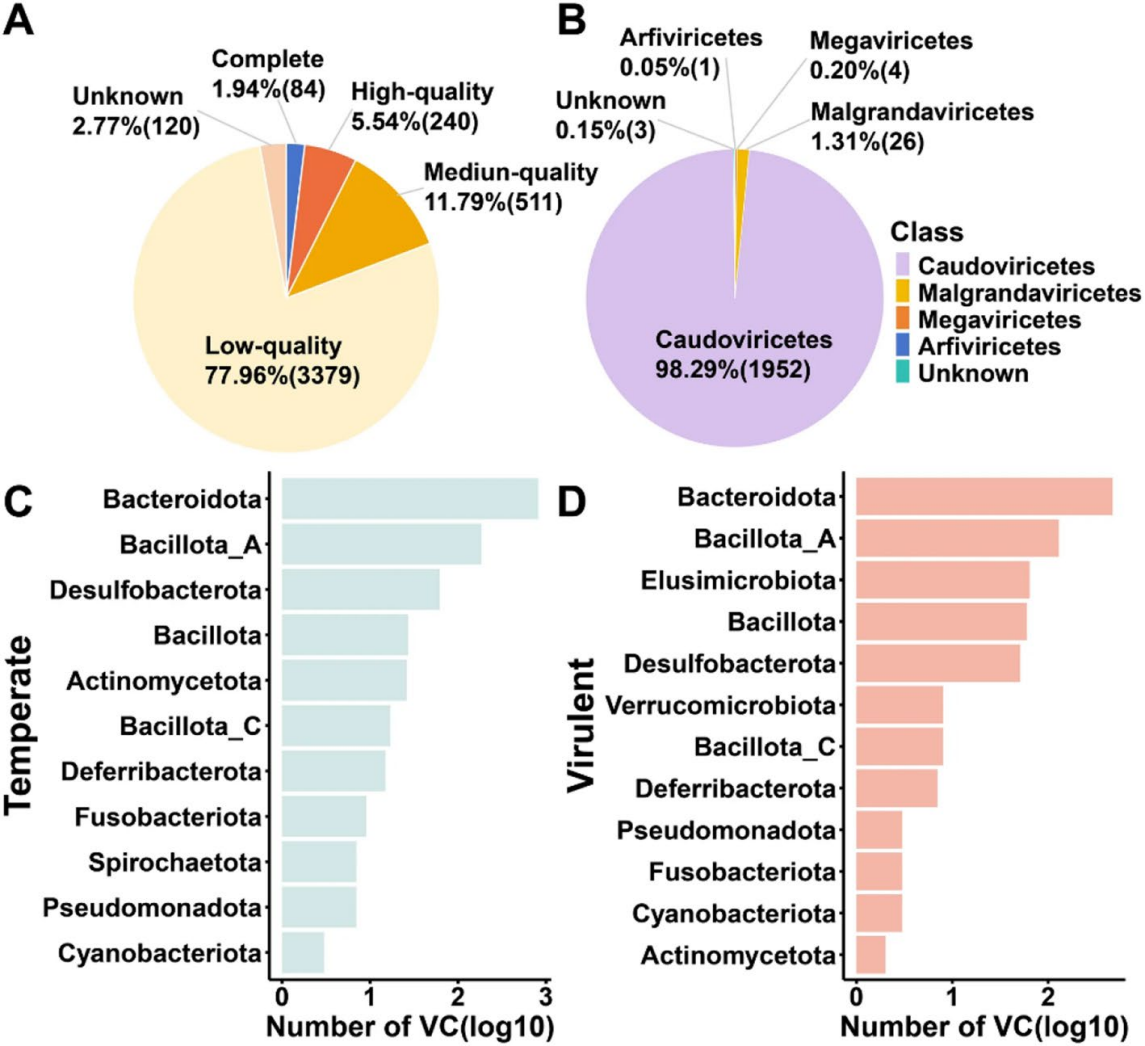
The viral contig characteristics and virus-host associations from 36 cecal metagenomes. **A** The distribution of the estimated completeness of 4,334 viral contigs. **B** The classification information of the class level of 1,986 vOTUs. **C**, **D** Number of temperate and virulent viral clusters (VCs) with > 50% completeness for each predicted host at the phylum level

Metagenomic assembly of 36 cecal samples yielded 4334 quality-filtered viral contigs (Fig. 3A). Among these, 835 high-confidence contigs (completeness ≥ 50%) had a mean length of 41,983 bp (N50 = 50,390 bp). Clustering at 95% average nucleotide identity (ANI) and 85% alignment fraction (AF) identified 1986 viral operational taxonomic units (vOTUs), including 428 with ≥ 50% completeness. All vOTUs represented phages, predominantly within *Caudoviricetes* (tailed phages), *Malgrandaviricetes* (microviruses), *Megaviricetes*, and *Arfiviricetes* (Fig. 3B). Protein-sharing clustering of high-quality vOTUs generated 392 viral clusters (VCs). Host prediction revealed a total of 1,984 virus-host linkages, comprising 808 lytic VC-host and 1176 temperate VC-host linkages. The hosts of temperate phages spanned across 11 phyla (Fig. 3C), whereas those of lytic phages were associated with 12 phyla (Fig. 3D). As expected, *Bacteroidota* (comprising 58.17% of lytic and 69.56% temperate viral hosts) and *Bacillota_A* (comprising 15.97% of lytic and 15.73% temperate viral hosts), highly predominant gut bacterial taxa, were the most common hosts of gut viruses at the phylum level. *Bacteroides* and *Phocaeicola* were the most prevalent hosts at the genus level (Fig. S3A, B). Among these VC-host linkages, VC2778, VC2779, and VC2563 exhibited the highest host diversity, each associated with 62, 54, and 49 distinct host species (Fig. S3C).

### Virome divergence and metabolic modulation in high- and low-FE Ducks

**Fig. 4:**
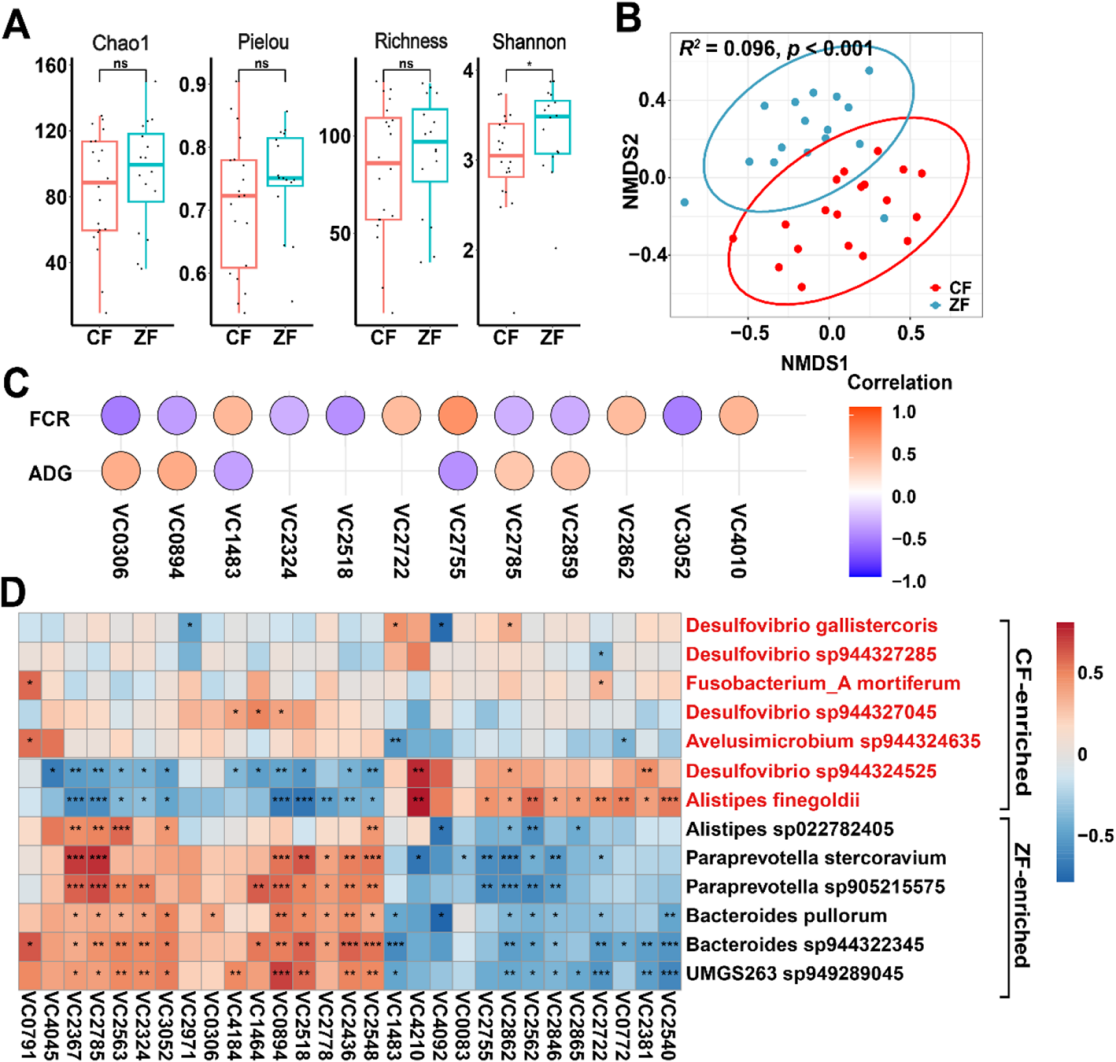
Viral diversity and their relationship with FE related traits. **A** Viral diversity (Chao1, Pielou, Richness, Shannon index) between two groups. **B** The NMDS plot of cecal viral communities between two groups based on Bray–Curtis dissimilarity. **C** Spearman’s rank correlation between FE traits and differential VCs, with color gradients representing positive (red) and negative (blue) correlation coefficients (*p* < 0.05). **D** Spearman’s rank correlation between differential VCs and differential bacteria (CF-enriched bacteria are colored red). ***p < 0.001, **p < 0.01, *p < 0.05

To compare gut viral communities between ZF and CF ducks, we examined viral α- and β-diversity of two groups. The results showed that the two groups exhibited similar α-diversity profiles except for the Shannon index at the VC level (Fig. 4A), while β-diversity analysis (NMDS, Bray–Curtis) revealed distinct clustering of individuals from the two groups (PERMANOVA, *R*^2^ = 0.096, *p* < 0.001; Fig. 4B).

To further investigate gut viral signatures associated with FE, we performed ANCOM-BC analysis and identified that 32 VCs were significantly decreased, while 19 VCs were increased in CF group compared with ZF group (Fig. S3D). Then we did the correlation analysis between these differential VCs and FE-related phenotypes. Twelve viruses (5 positive and 7 negative) and 6 viruses (4 positive and 2 negative) displayed significant correlations with FCR and ADG, respectively (Spearman, |*r*|≥ 0.3, *p* < 0.05) (Fig. 4C). For example, VC2859, VC2785, and VC0894 showed consistent positive ADG or negative FCR correlations. As phages may link to phenotype by modulating the composition and dynamics of bacterial communities, we predicted interactions of these FE-associated VCs (either co-occurrence or mutual exclusion) with differentially abundant bacteria (Fig. 4D). Spearman correlation analysis revealed distinct phage-bacteria association patterns. One group of VCs, including VC2785, VC0894, and VC2518, displayed significant positive correlations (co-occurrence) with high-FE favored bacteria (e.g., *Paraprevotella sp905215575*, *Bacteroides sp944322345*, *UMGS263 sp949289045*) (*r* = 0.41 ~ 0.67, *p* < 0.05), while exhibiting significant negative correlations (mutual exclusion) with low-FE favored bacteria (e.g., *Alistipes finegoldii*, *Desulfovibrio sp944324525*) (*r* = − 0.70 ~ − 0.47, *p* < 0.05). Conversely, a second group of VCs (e.g., VC2862, VC2755, VC2722, VC1483) displayed the opposite association pattern, positively correlating with low-FE favored bacteria and negatively correlating with high-FE favored bacteria. Importantly, to distinguish ecological interactions from direct infection, we cross-referenced these correlations with our in silico host predictions. We found that key strong positive correlations, such as that between VC2785 and *Paraprevotella*, were concordant with predicted phage-host linkages, reinforcing the biological validity of these associations.

**Fig. 5:**
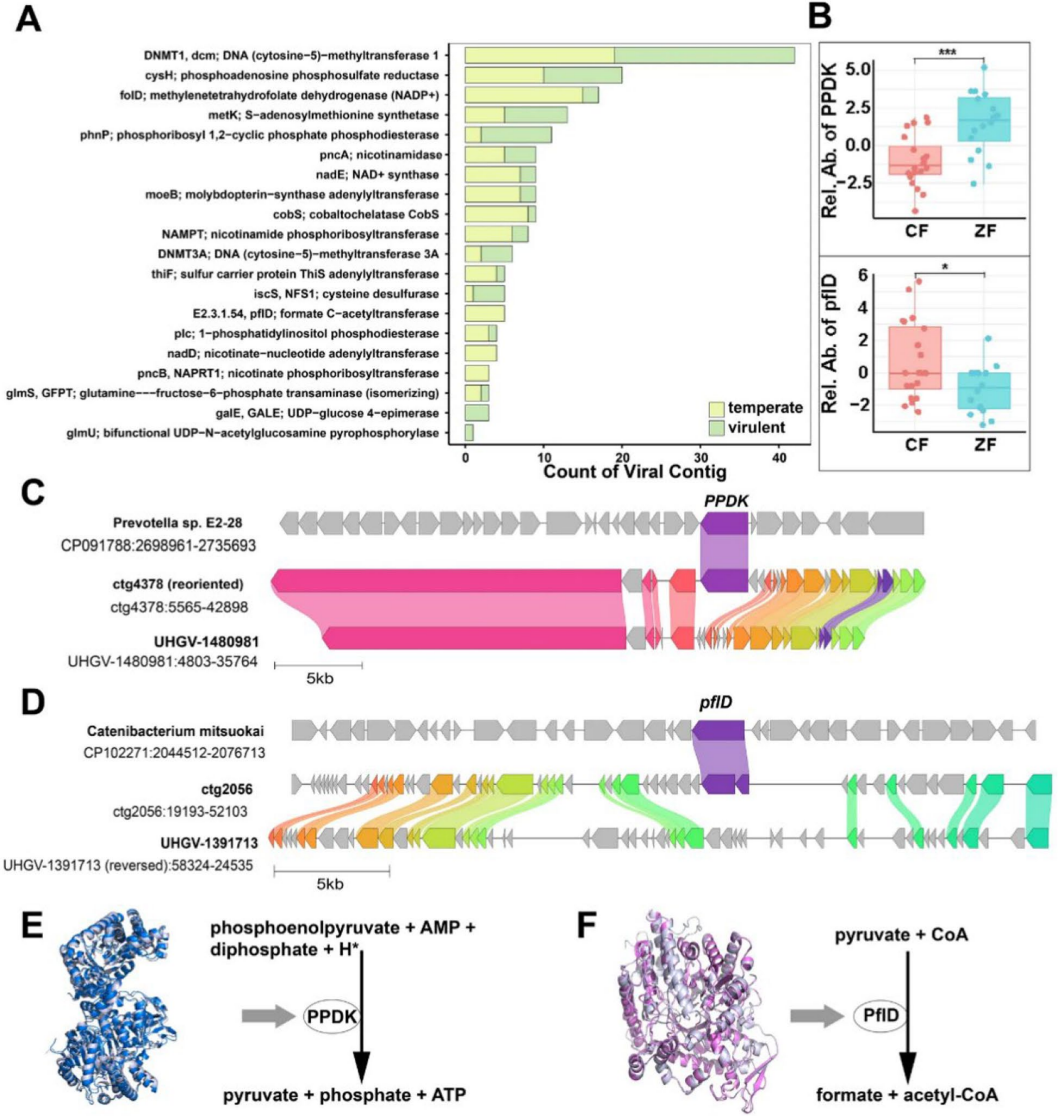
Auxiliary metabolic genes (AMGs) carried by duck cecal viruses. **A** A bar plot showing the occurrence (log10) of AMGs identified in the temperate (green) and virulent (yellow) viruses. **B** The relative abundance of two AMGs (PPDK and pflD) in two groups. ***p < 0.001, *p < 0.05. **C** Genome synteny maps of two viruses (ctg4378 is identified in this study) and one bacterium containing the PPDK gene. **D** Genome synteny maps of two viruses (ctg2056 is identified in this study) and one bacterium containing the pflD gene. Homologous genes from different organisms are filled in the same color and connected using the shading links. **E** Predicted tertiary structure and function of the phage encoded PPDK protein. The phage-encoded PPDK structure is superimposed onto the reference structure of PPDK from Clostridium symbiosum (UniProt: P22983; colored silver) based on Foldseek search [24]. PPDK (EC 2.7.9.1) catalyzes the conversion of AMP, phosphoenolpyruvate, and diphosphate to ATP, pyruvate, and inorganic phosphate. **F** Predicted tertiary structure and function of the phage encoded pflD protein. The phage-encoded pflD structure is superimposed onto the reference structure of pflD from Enterococcus faecium (UniProt: A0A132P2N7; colored silver) based on Foldseek search. pflD (EC 2.3.1.54) catalyzes the conversion of pyruvate and CoA to formate and acetyl-CoA

Then we searched AMGs from viral contigs and found 109 AMGs that were involved in a wide range of metabolic processes, including the metabolism of nucleotides, carbohydrates, amino acids, and sulfur (Fig. 5A). The most prevalent AMG was DNMT1, a DNA (cytosine-5)–methyltransferase that prevents viruses from being recognized and cleaved by the restriction-modification systems of their hosts [22, 23], which were detected in more than 40 high-quality viral contigs. CysH was the second highly abundant AMG, which encodes a phosphoadenosine phosphosulfate reductase involved in the synthesis of sulfite from sulfate. Additionally, another abundant AMG, metK encoding S-adenosylmethionine (SAM) synthetase, was important for genomic DNA methylation and cell division. Two AMGs, NAMPT and pncA, were implicated in NAD + metabolism, which is critical for maintaining energy homeostasis. CobS and cobT were two key AMGs involved in the synthesis of vitamin B12.

We subsequently conducted a differential analysis of AMGs between CF and ZF groups and found 16 AMGs showing significant differential abundance. Notably, two differential AMGs, pyruvate phosphate dikinase (PPDK) and formate C-acetyltransferase (pflD), involved in pyruvate metabolism were identified in viral contigs (Fig. 5B). This PPDK shared 83% amino acid identity with PPDK in *Prevotella sp. E2-28* (GenBank: CP091788) (Fig. 5C), while the pflD was 52% identical to a pflD in *Catenibacterium mitsuokai strain DSM 15897* (GenBank: CP102271) (Fig. 5D). Specifically, in the anaerobic cecal environment, temperate virus (VC2785)-encoded PPDK, catalyzing the reversible conversion of phosphoenolpyruvate (PEP) to pyruvate and ATP [25], was higher abundant in the ZF group compared with the CF group, which may augment the fermentative capacity of host bacteria (e.g., *Bacteroides* and *Paraprevotella*) in the ZF group (Fig. 5E). This is in line with the abovementioned observation that VC2785 displayed a positive correlation with both ADG (Fig. 4D). While lytic virus (VC0717)-encoded pflD, converting pyruvate to acetyl-CoA and formate, was higher abundant in the CF group compared with the ZF group, which may accelerate viral replication by hijacking the host’s metabolic machinery in the CF group (Fig. 5F). Moreover, its host, *Prevotella sp015074785*, belonging to the *Prevotellaceae* family, significantly contributes to the production of SCFAs, which may induce lytic viral replication and lysis of cells [26, 27]. These results together suggest that gut viruses could potentially optimize nutrient availability for their hosts or exploit host central metabolic pathways for their own replication, underscoring their influence on host physiology including FE.

### Serum metabolome profiling reveals systemic metabolic divergence between two groups

**Fig. 6:**
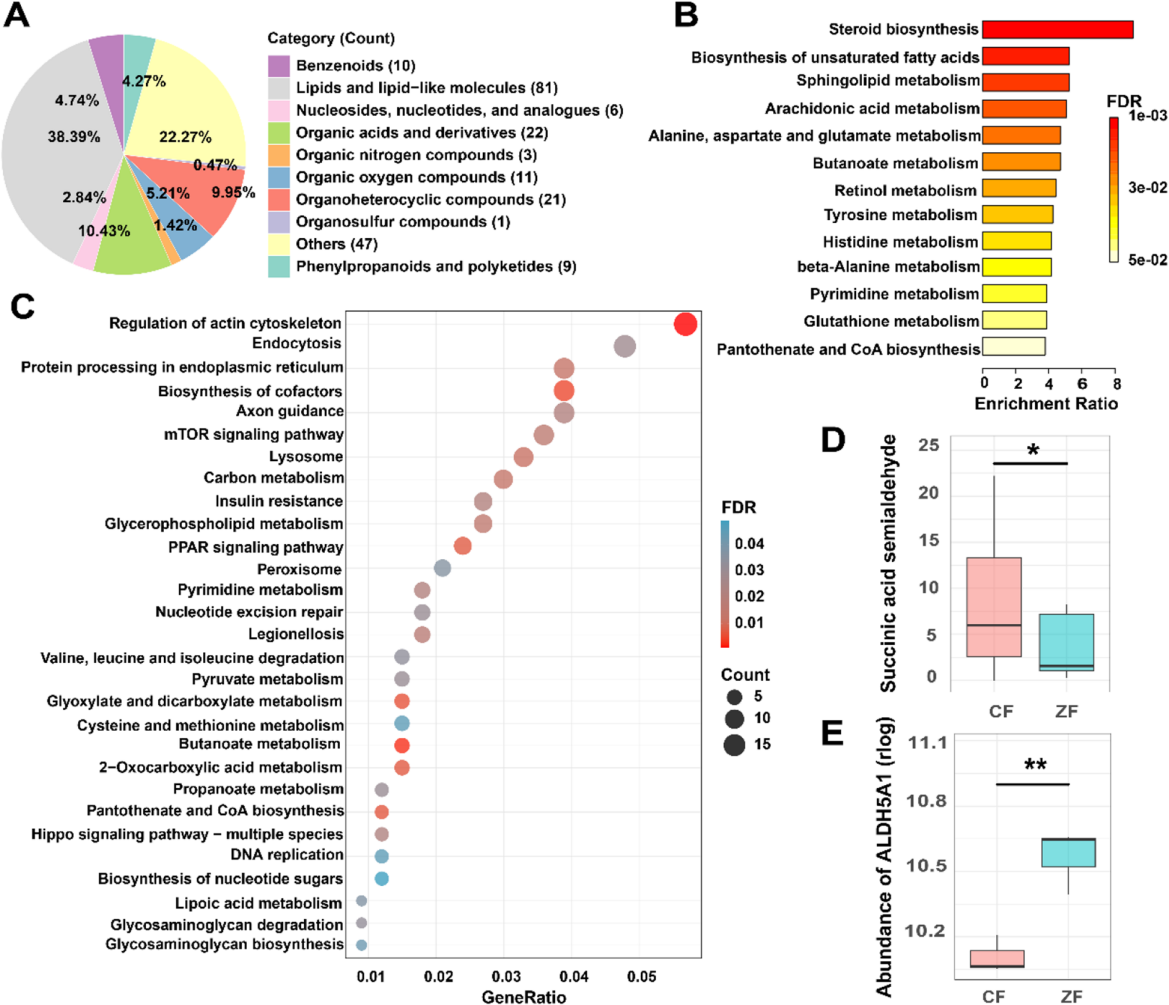
Serum metabolome and liver transcriptome profiles reveal systemic metabolic divergence between the two groups. **A** Classification and composition of differentially abundant serum metabolites between two groups. **B** Functional enrichment analysis of differentially abundant metabolites and the resulting *p*-values were adjusted for multiple testing using the Benjamini–Hochberg FDR method. **C** KEGG pathway enrichment analysis of differentially expressed genes (DEGs) in the liver between two groups and the resulting *p*-values were adjusted for multiple testing using the Benjamini–Hochberg FDR method. **D** Relative abundance of succinic semialdehyde in two groups. **E** Expression level of succinic semialdehyde dehydrogenase (ALDH5A1) in two groups. **p < 0.01, *p < 0.05

Serum metabolomics identified 211 differentially abundant metabolites between CF and ZF ducks (|log_2_FC|≥ 1, *p* < 0.05), with 175 upregulated and 36 downregulated in CF compared with ZF (Fig. S4). According to the annotation of the Human Metabolome Database (HMDB), these differential metabolites were mainly categorized into lipids and lipid-like molecules (38.39%), organic acids and derivatives (10.43%), organoheterocyclic compounds (9.95%), and organic oxygen compounds (5.21%) (Fig. 6A). Metabolite pathway enrichment analysis indicated that these differential metabolites were mainly enriched in 13 pathways (Fig. 6B). Among them, 3 pathways including “pyrimidine metabolism”, “butanoate metabolism”, and “pantothenate and CoA biosynthesis” showed concordant enrichment in liver transcriptomes (Fig. 6C). For example, in butanoate metabolism, we observed higher abundance of succinic semialdehyde in the CF group compared with the ZF group (log_2_FC = 1.37, *p* < 0.05); this may stem from downregulation of the succinic semialdehyde dehydrogenase (ALDH5A1) gene (log_2_FC = − 0.75, *p* < 0.01), a mitochondrial homotetramer protein that catalyzes the NAD + -dependent conversion of succinic semialdehyde into succinate, which can be further fermented to butanoate (Fig. 6D, E). In functional enrichment analysis of DEGs, we also observed multiple pathways related to lipid metabolism, such as “insulin resistance”, “PPAR signaling pathway”, and “glycerophospholipid metabolism”, explaining the predominant lipid metabolite differences observed between the two groups (Fig. 6C).

### Gut microbiome-serum metabolome associations with feed efficiency

**Fig. 7:**
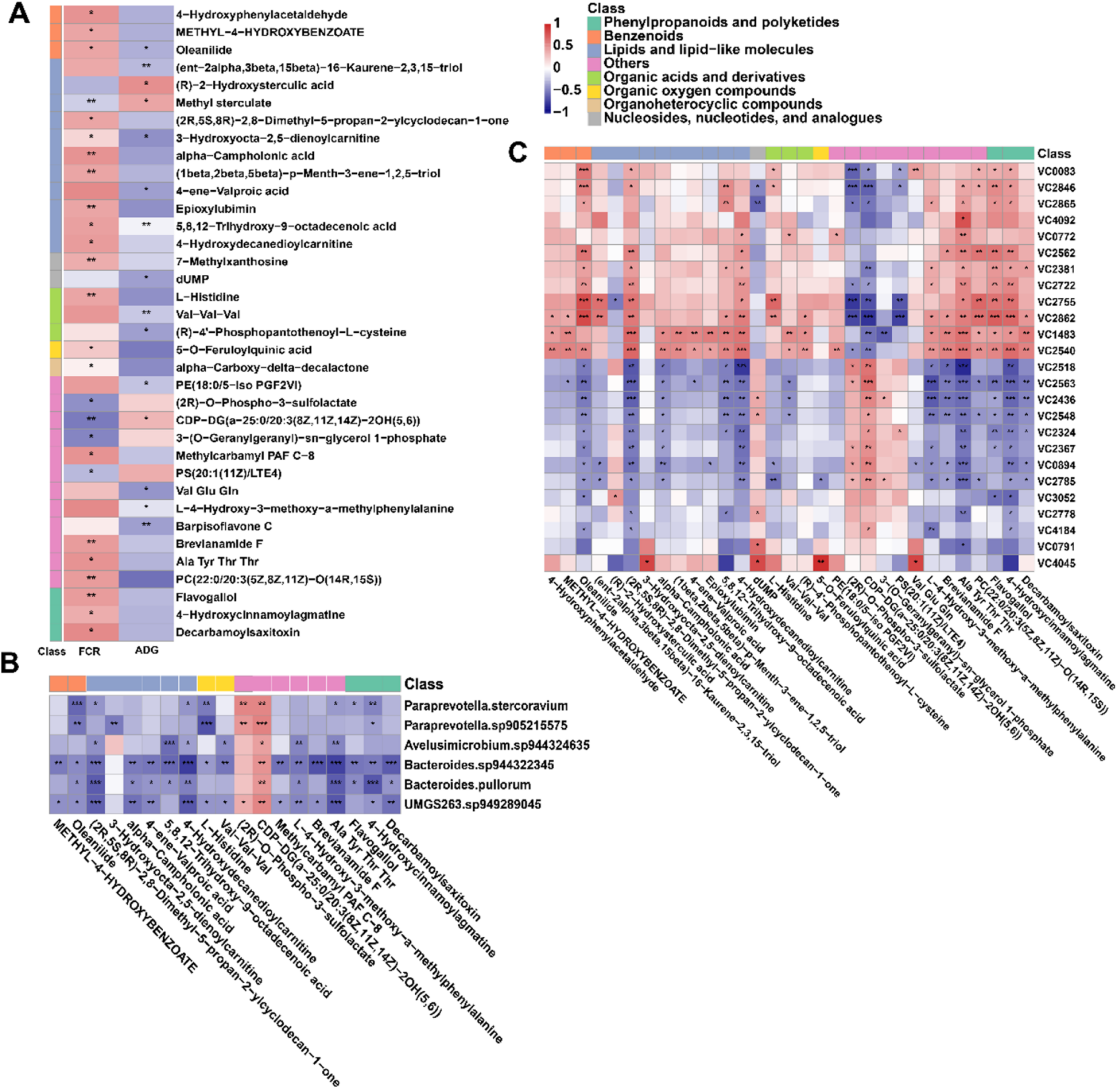
Correlations between gut microbiome and serum metabolome. **A** Spearman’s rank correlations between differential serum metabolites and FE traits. **B** Spearman’s rank correlations between FE-associated metabolites and differentially abundant bacteria. **C** Spearman’s rank correlations between FE-associated metabolites and differential viruses. Metabolites are clustered by their biochemical classifications. ***p < 0.001, **p < 0.01, *p < 0.05

Among differential serum metabolites, 36 metabolites were significantly correlated with FE traits (|*r*|≥ 0.3, *p* < 0.05), with most of them positively correlated with FCR (21/36) (Fig. 7A). For example, L-histidine and 4-hydroxydecanedioylcarnitine were negatively correlated with ADG, while positively associated with FCR. To detect the potential effect of differentially abundant bacteria on host FE-associated metabolites, spearman’s rank correlations revealed a total of 67 strong correlations (|*r*|≥ 0.5, *p* < 0.05; Fig. 7B). Among these correlations, most of them were negative between differential bacteria and FE-associated metabolites (e.g., *Paraprevotella sp905215575* vs. L-histidine: *r* = − 0.67), while these bacteria were positively correlated with two metabolites (e.g., *UMGS263 sp949289045* vs. CDP-DG (a-25:0/20:4(6E,8Z,11Z,14Z) + = O(5)): *r* = 0.48). Spearman’s rank correlations between differential viruses and FE-associated metabolites were also performed, most FE-associated metabolites showed positive correlations with 12 VCs (e.g., VC2540 vs. 4-hydroxydecanedioylcarnitine: *r* = 0.64) and negative correlations with 13 VCs (e.g., VC2785 vs. L-histidine: *r* = − 0.56), respectively, while the correlation of CDP-DG(a-25:0/20:4(6E,8Z,11Z,14Z) + = O(5)) and (2R)-O-Phospho-3-sulfolactate with VCs showed the reversed pattern (Fig. 7C).

## Discussion

In the past decade, gut microbiome and its associated roles have attracted increasing attention. Although several studies have investigated the bacterial contributions to FE of poultry using 16S rRNA sequencing [8, 28], the role of gut virome remains poorly characterized. Our multi-omics integration (metagenomics, transcriptomics, metabolomics) reveals associations between bacterial and viral components of the duck gut microbiome and FE-associated phenotypes.

We identified that ZF ducks have higher FE traits and cecal SCFAs (e.g., propionate and butyrate) concentration compared with CF group. Due to pivotal roles of SCFAs in maintaining energy homeostasis and promoting animal health through multifaceted mechanisms, including maintenance of intestinal barrier integrity and improvement of nutrient absorption [29], thus animals producing more SCFAs usually may have high FE. Previous studies showed that butyrate supplementation improved FE in chicken and duck [30, 31]. In a sheep study, higher concentration of propionate was also detected in rumen of high FE group [9].

In duck, SCFAs are chiefly produced in the cecum; thus, the difference of SCFAs between two groups may arise from variation in cecal bacterial diversity, composition, and function. We found that cecal bacteria in high-FE (ZF) and low-FE (CF) ducks shared core phyla (*Bacteroidetes* and *Bacillota*) but exhibited divergent diversity and network architectures. ZF microbiomes displayed higher modularity (Fig. S2G, H), indicative of more persistent and stable community-level and within-community interactions [32, 33]. Besides network topology, the relative abundances of bacteria at various taxonomic levels in the two groups also exhibit differences; most of the differential species are enriched in the ZF group. Crucially, these ZF-enriched bacteria (e.g., *Paraprevotella sp905215575* and *Bacteroides sp944322345*) potentially drove efficient pyruvate flux toward SCFA production rather than methanogenesis (Fig. 4E, F). This is consistent with the observation of elevated cecal propionate/butyrate in ZF ducks. It has been reported that *Paraprevotella* was more enriched in the leaner pigs [34], which usually have high FE traits [8, 35]. Conversely, CF-enriched *Desulfovibrio sp944324525* and *Alistipes finegoldii* correlated negatively with SCFAs (Fig. S2H), aligning with inefficient energy harvesting; this was also observed in chicken gut [36].

As the “dark matter” of the gut microbiome, phages (predominantly *Caudoviricetes*) can alter the bacterial community structure by lysing certain bacteria or through lysogeny, thereby affecting animal phenotypes. There was a significant difference in β diversity of viruses, suggesting a distinct viral community structure in two groups. In addition, multiple differentially abundant viruses showed a significant correlation with FE traits in ducks. From the predicted phage–host linkages, we found that some FE-correlated phages could target SCFA producers, such as *Paraprevotella sp905215575* and *Bacteroides sp944322345*, which are favorable for high FE. These results position phages as key phenotype mediators, which is supported by our human gut findings [14].

During the phage infection of bacteria, AMGs carried by phages play crucial roles in optimizing the host’s metabolism and nutrient acquisition, which affect several important ecological processes, including methane metabolism in different habitats [37], polysaccharide digestion in the ruminants [12], and SCFAs metabolism in the human gut [14]. However, the contribution of viral AMG in poultry gut has not been reported yet. Our results identified a total of 109 AMGs in duck cecum, which participated multiple processes, such as antiviral defense (e.g., DNMT1), sulfur metabolism (e.g., cysH and metK), energy homeostasis (e.g., NAMPT and pncA), and vitamin biosynthesis (cobS and cobT), suggesting that the gut virome can also affect feed digestibility in duck. We identified a total of 16 differentially abundant AMGs between two groups, with two AMGs, PPDK and pflD, involved in pyruvate metabolism process. In anaerobic environments like the cecum, microbes rely on fermentation pathways. PPDK enables the regeneration of PEP, which serves as a key intermediate for substrate-level phosphorylation, a primary ATP-generating mechanism under anaerobic conditions [25]. Given the requisite integration of temperate phages into the host genome, the temperate phages-encoded PPDK enriched in ZF potentially boosted pyruvate metabolism and SCFAs production of host bacteria, thereby conferring communal benefits and, in turn, reciprocal advantages to the viruses themselves [38]. While the lytic phage-encoded pflD enriched in CF could hijack host resources to produce more acetyl-CoA (SCFAs), which may promote viral infection and host lysis [39]. However, the underlying mechanisms remain to be explored and need further experimental validation.

Gut microbiota produce a variety of molecules, some of which are taken up into the bloodstream and affect host health. Therefore, characterizing the interactions between gut microbiota and host plasma metabolites is also important for understanding the effects of the gut microbiota on animal phenotypes. Our results revealed that L-histidine and succinic semialdehyde were more abundant in serum of the CF group compared with the ZF group. Consistently, recent papers have reported that plasma L-histidine was downregulated in lambs with high ADG [40, 41]. Besides, our RNA-seq data revealed reduced hepatic ALDH5A1 expression in CF ducks, which could impair succinic semialdehyde → butanoate conversion and lead to accumulation of succinic semialdehyde (Fig. 7D). Intriguingly, succinic semialdehyde levels were found to be significantly positively correlated with insulin resistance and liver fat content [42], concordant with dysregulated lipid pathways, such as “insulin resistance”, “PPAR signaling pathway”, and “glycerophospholipid metabolism”, in the CF group.

We reported that these serum FE-related metabolites were associated with duck cecal microorganisms, where most of them displayed strong negative associations with multiple differential bacterial and viral species between groups. The negative correlations of plasma L-histidine level with *Bacteroides* and *Paraprevotella* are in harmony with the results observed in piglets, rats, and fish [43–45]. This is probably due to the contribution of *Bacteroides* and *Paraprevotella* genera to L-histidine degradation [46, 47]. Due to “virus-host” interactions, where viruses lyse host bacteria or boost their metabolism, gut viruses also display correlations with plasma metabolites. However, these links still require experimental validation in further study. Such information will provide evidence highlighting the possibility of animal blood metabolites through manipulating gut microbial functions.

## Conclusion

**Fig. 8:**
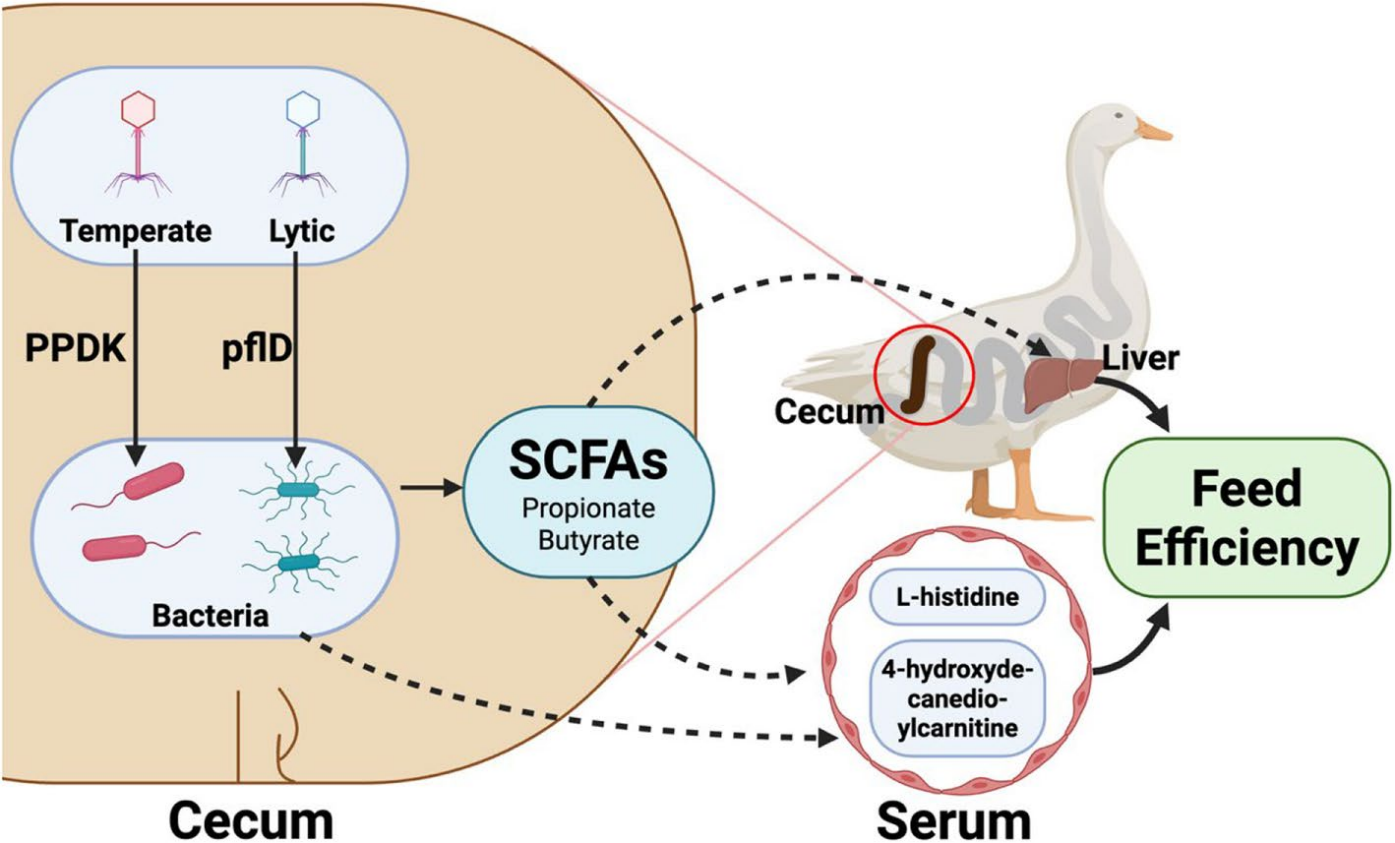
The potential mechanism by which bacteria and phage regulate cecal SCFA production and host metabolism to improve FE in ducks. Phages modulate bacteria to produce SCFAs through encoded AMGs, PPDK, and pflD

Taken together, to elucidate the mechanisms by which gut microbiota affect FE of ducks, we proposed two potential convergent pathways: (1) SCFAs are the major end products from the fermentation of gut bacteria. Viruses directly target SCFA-producing bacteria, altering community function and output, thereby jointly impacting FE through collaborative interactions with bacteria; (2) gut bacteria and viruses interact to modulate host animal metabolism via blood circulation and thus influencing FE (Fig. 8). However, further studies are required to validate the actual transcriptional activity, function of phage-encoded AMGs, and causal relationships between representative SCFA-producing bacteria and phage consortia and host physiological outcomes.

## Materials and methods

### Animals management

Two Pekin duck populations with divergent FE were used: a high-FE group (ZF) and a low-FE group (CF). A total of 300 1-day-old male ducklings (150 per group) with similar initial body weights were housed individually in wire-floor cages (200 × 100 × 40 cm) equipped with nipple drinkers and tubular feeders at the National Changping Comprehensive Agricultural Engineering and Technology Research Center (Livestock and Poultry Sub-Center). Ducks were reared in two phases (days 1–14 and days 15–42) according to standardized practices. Both groups received identical commercial diets (Hope Feed Co., Ltd., Fangshan District, Beijing, China); diet composition and nutritional components matched those described in our previous study [48].

### Feed efficiency measurement and sample collection

Body weight (BW) was recorded at 22 and 42 days of age. Total feed intake (FI) was measured from 22 to 42 days. Average daily feed intake (ADFI), average daily gain (ADG), and feed conversion ratio (FCR) were calculated as follows:

1 $$\mathrm{ADFI} (\mathrm{g}/\mathrm{day}) = \text{Total FI}/21\ \mathrm{days}$$

2 $$\mathrm{ADG} (\mathrm{g}/\mathrm{day}) = (\mathrm{BW_{42}}-\mathrm{BW_{22}})/21 \; \mathrm{days}$$

3 $$\mathrm{FCR} = \mathrm{Total} \mathrm{FI}/(\mathrm{BW_{42}}-\mathrm{BW_{22}})$$

Valid phenotypic records were obtained for 283 ducks. After a 12-h fast, ducks were humanely euthanized according to standard commercial protocols. Fatness traits were recorded, including skin fat weight (SFW), skin fat percentage (SFP), abdominal fat weight (AFW), and abdominal fat percentage (AFP), calculated as.

4 $$\mathrm{SFP} (\%) = (\mathrm{SFW} / \text{carcass weight}) \times 100$$

5 $$\mathrm{AFP} (\%) = (\mathrm{AFW} / \text{carcass weight}) \times 100$$

### DNA extraction, 16S amplicon and metagenomic sequencing

Ducks with valid FCR records are euthanized by cervical dislocation and dissected. Cecal contents (including chyme and mucosa) were immediately collected, snap-frozen in liquid nitrogen, and stored at − 80 °C until further processing.

Microbial DNA was extracted from cecal samples of 283 ducks using the QIAamp DNA Stool Mini Kit (QIAGEN, Hilden, Germany). The V3–V4 hypervariable region of the bacterial 16S rRNA gene was amplified using primers 338 F (5′-ACTCCTACGGGAGGCAGCA-3′) and 806R (5′-GGACTACHVGGGTWTCTAAT-3′). Amplified libraries were sequenced (2 × 250 bp) on an Illumina NovaSeq 6000 platform.

A subset of 40 ducks (20 CF, 20 ZF) was randomly selected. Microbial DNA was extracted from cecal content using a CTAB-based method. DNA quality and quantity were assessed using a Qubit 3.0 Fluorometer (Invitrogen). Four ZF samples were excluded due to insufficient DNA yield, resulting in 36 samples (20 CF, 16 ZF) for library preparation. Libraries were constructed using the VAHTS Universal Plus DNA Library Prep Kit (Illumina, San Diego, CA, USA) and sequenced (2 × 150 bp) on an Illumina NovaSeq X platform following standard protocols.

### SCFA concentration measurement

Cecal samples from the 36 ducks used for metagenome sequencing were also analyzed for the concentration of 10 SCFAs: acetic acid, propionic acid, isobutyric acid, butyric acid, isovaleric acid, valeric acid, hexanoic acid, heptanoic acid, nonanoic acid, and decanoic acid. Briefly, samples were thawed on ice. Approximately 0.5 g of cecal content was homogenized in 1 mL of deionized water for 4 min at 40 Hz, followed by ultrasonic oscillation for 15 min on ice. The homogenate was centrifuged at 5000 rpm for 20 min at 4 °C. A 0.8-mL aliquot of the supernatant was mixed with 0.1 mL of 50% (v/v) sulfuric acid (H_2_SO_4_) and 0.8 mL of extraction solution containing 25 mg/L internal standard (methyl tert-butyl ether, MTBE). The mixture was vortexed vigorously for 10 min and centrifuged at 10,000 rpm for 15 min at 4 °C. The resulting supernatant was analyzed using a SHIMADZU GC2030-QP2020 NX gas chromatography–mass spectrometer (GC–MS). Absolute SCFA concentrations were determined using external calibration curves generated from standard solutions of each SCFA at varying concentrations.

### Determination and analysis of plasma metabolome

Plasma metabolites were analyzed using ultra-high-performance liquid chromatography (UHPLC) coupled with tandem mass spectrometry (MS/MS). Chromatographic separation was performed on an ACQUITY UPLC® HSS T3 column (100 mm × 2.1 mm, 1.8 μm average particle size, Waters Corporation) using 0.1% (v/v) formic acid aqueous solution and acetonitrile as mobile phases. To mitigate analytical bias from instrumental variation, a pooled quality control (QC) sample was created by combining equal aliquots of all sample supernatants. This QC sample was injected at regular intervals throughout the analytical sequence. Raw MS data were acquired using MassLynx™ V4.2 software (Waters Corporation). Data processing—including peak detection, alignment, and integration—was performed in Progenesis QI (Nonlinear Dynamics). Metabolite annotation was conducted against the METLIN database (https://metlin.scripps.edu) within the Progenesis QI platform, using a mass accuracy threshold of ≤ 10 ppm and MS/MS spectral matching.

### Preparation of libraries for RNA-sequencing and data analysis

Liver tissues were collected from three CF and three ZF ducks immediately post-euthanasia, snap-frozen in liquid nitrogen, and stored at − 80 °C. Total RNA was extracted using TRIzol™ Reagent (Thermo Fisher Scientific, Waltham, MA) following manufacturer protocols. RNA integrity was assessed by UV spectrophotometry (NanoDrop™ 2000; Thermo Fisher Scientific) and Electropherogram analysis (Agilent 2100 Bioanalyzer; Agilent Technologies, Santa Clara, CA, USA). Ribosomal RNA was depleted using the Illumina TruSeq Stranded Total RNA Library Prep Kit with Ribo-Zero Gold. Resulting mRNA was fragmented and reverse-transcribed into cDNA libraries using the same kit. Libraries were sequenced (2 × 150 bp) on an Illumina HiSeq X platform.

Raw reads were quality-trimmed and adapter-filtered using fastp (v0.20.0) [49]. Processed reads were mapped to the Pekin duck reference genome (ZJU1.0) using STAR (version 2.7.9a) [50] with default parameters. Gene-level counts were generated with STAR’s –quantMode GeneCounts option and analyzed for differential expression using DESeq2 (v1.26.0) [51] in R/Bioconductor, with normalization via the median-of-ratios method.

### 16S amplicon sequencing data processing

Raw sequencing reads underwent quality assessment using FastQC. Adapters and low-quality bases were trimmed using fastp (v0.20.0). Processed reads were denoised, error-corrected, and filtered for chimeras using the DADA2 plugin (v1.18) [52] in QIIME2 (v2020.6) [53], generating amplicon sequence variants (ASVs). ASVs were filtered to retain those with relative abundance > 0.0001% (10⁻^6^) and presence in ≥ 2 samples. Taxonomy was assigned against the Genome Taxonomy Database (GTDB r207) using DADA2’s native Bayesian classifier with default parameters. Alpha diversity (Shannon, Chao1, Pielou, and Richness) metrics were calculated in QIIME2. Phyloseq (v1.34.0) [54] was used for evaluation of sequencing depth and preparation of final ASV tables for downstream statistical analysis.

### Metagenome-assembled genomes (MAGs) recovery and annotation

Raw metagenomic reads were preprocessed using fastp (v0.20.0) for adapter trimming and quality filtering. Host-derived reads were removed by alignment to the duck reference genome (ZJU1.0) using Bowtie2 (v2.4.2) [55]. De novo assembly of filtered reads was performed per sample using metaSPAdes (v3.15.0) [56] with default parameters. Assembled contigs were subsequently binned into MAGs using an integrative approach with MetaBAT2 (v2.17) [57], MaxBin2 (v2.2.7) [58], and CONCOCT (v1.1.0) [59]. Consensus bins were generated and refined using DAS Tool (v1.1.3) [60]. The quality of the refined MAGs was assessed using CheckM2 (v1.0.1) [61] with default parameters. Taxonomic classification was performed against GTDB r207 using GTDB-Tk (v2.1.0) [62]. Community-level functional annotations were performed using HUMAnN 3.0 (v3.6) pipeline with the clean reads as input [63]. Species-level differential abundance between FE groups was determined using ANCOM-BC [64]. Open reading frames (ORFs) were predicted from all contigs using Prodigal (v2.6.3) [65] in metagenomic mode. Proteins were annotated using eggNOG database with eggNOG-mapper v2 [66]. A non-redundant gene catalog was constructed with CD-HIT (v4.8.1) [67] at 95% nucleotide identity and 90% coverage. Gene abundances were quantified by aligning reads to the non-redundant gene catalog using Salmon (v1.10.2) [68] in alignment-based mode. Differential abundance of KEGG Orthologs (KOs) was tested using MaAsLin2 (v1.12.0) [69] with default parameters and significance was determined based on *p*-value < 0.05, followed by FDR correction for pathway enrichment analysis.

### Viral contig identification and annotation

Putative viral contigs were first filtered from assembled metagenomic contigs using geNomad (v1.8.0) [70] with a relaxed threshold of –min-score = 0.75 to increase detection sensitivity. The putative viral contigs were annotated using our previously developed ViroProfiler pipeline [71] for precise viral contig identification and annotation. Briefly, The ViroProfiler pipeline utilizes CheckV (v0.9.0) [72] to remove host region in provirus contigs, and then use VirSorter2 (v2.2.4) [73] and VIBRANT (v1.2.1) [74] to identify viral contigs. Auxiliary metabolic genes (AMGs) were annotated using VIBRANT and DRAM-v (v1.4.4) [75]. Viral hosts were predicted using iPHoP (v1.2.0) with confidence score ≥ 90 [76]. Taxonomic classification was performed using a combination of geNomad and VITAP (v1.7.1) [77]. VITAP taxonomy was preferred due to its more specific annotations, often to genus or species level with high precision, while geNomad taxonomy was used when a contig was not annotated by VITAP. Viruses were classified as temperate if they are identified as provirus by geNomad, VIBRANT, or CheckV (v0.9.0), or classified as temperate phages by BACPHLIP (v0.9.6) [78]. Viral contigs were then dereplicated to generate a non-redundant catalog of viral sequences. Specifically, pairwise average nucleotide identity (ANI) was calculated among these contigs using the clustering algorithm within CheckV, applying thresholds of ≥ 95% ANI and ≥ 85% AF to define species-level viral operational taxonomic units (vOTUs). The longest contig within each vOTU cluster was selected as its representative sequence. To further investigate relationships among the identified vOTUs, compare them with known viral genomes, and identify potential novel viral clusters (VCs), gene-sharing network analysis was conducted using vConTACT2 (v.0.11.3) [79]. VCs identified by vConTACT2 represent groups of related viral genomes, offer insights into potential genus-level relationships, and highlight vOTUs that may constitute novel taxa. Abundance of viruses were estimated by mapping clean reads to vOTUs using Bowtie2, and then quantified using CoverM (v0.7.0) (https://github.com/wwood/CoverM). For all downstream analyses, including diversity calculations, differential abundance testing, host prediction, and AMG identification, only vOTUs with an estimated completeness ≥ 50% were used.

### Statistical analyses

All statistical analyses were performed in R (v4.1.0). Data manipulation and visualization utilized the tidyverse ecosystem (v1.3.0). Student’s *t*-test and Wilcoxon rank sum test were employed for group comparisons. The analysis of differentially abundant bacterial species and viral clusters (VCs) was conducted using ANCOM-BC with Benjamini–Hochberg False Discovery Rate (FDR) method. For transcriptome data, differentially expressed genes (DEGs) were identified using DESeq2 with thresholds of adjusted *p*-value (Benjamini-Hochberg) < 0.05 and |log_2_FC|≥ 0.585. KEGG pathway enrichment of DEGs was performed using ClusterProfiler (v4.0) [80] and the resulting *p*-values were adjusted for multiple testing using the FDR correction. For metabolomics data, peak areas were normalized via total sum scaling. Prior to differential abundance analysis, metabolites that were undetected (missing) in more than 50% of samples within both groups were excluded to prevent statistical artifacts and artificial fold-change inflation. Subsequently, differential metabolites between two groups were identified with threshold of |log_2_**FC**|≥ 1 and *p* < 0.05. Metabolic pathway enrichment was analyzed in MetaboAnalyst 5.0 (https://www.metaboanalyst.ca) with FDR correction.

## Supplementary Information

Supplementary Material 1: Supplementary Figure S1: FE traits and SCFAs concentrations between two groups. Supplementary Figure S2: Bacterial composition, network analysis, and functional correlations between two groups. Supplementary Figure S3: Host association and differential abundance of viral clusters (VCs). Supplementary Figure S4: Volcano plot of differentially abundant metabolites in CF vs. ZF. Supplementary Table S1: Growth, feed efficiency and fatness traits in two groups. Supplementary Table S2: Comparative topological properties of the CF and ZF networks. Supplementary Table S3: Degree and eigenvector centrality of the hub nodes in CF and ZF networks.

## Data Availability

The raw data for multi-omics cohort are deposited in the National Genomics Data Center website (https://ngdc.cncb.ac.cn). All sequencing data are accessible with the identifier CRA026543 (amplicon), CRA028005 (metagenome), and CRA027933 (transcriptome). The metabolomics data are accessible with the identifier OMIX011117. The code used for data analysis is available at the GitHub repository https://github.com/rujinlong/duckbiome.
