# Supplementary material for: Bacteria and phage consortia modulate cecal SCFA production and host metabolism to enhance feed efficiency in ducks
Source: Microbiome. 2026 Mar 4;14:113. doi: 10.1186/s40168-026-02368-y (PMC13069815; doi:10.1186/s40168-026-02368-y)
Supplement: Supplementary file 2 — Supplementary Material 1: Supplementary Figure S1: FE traits and SCFAs concentrations between two groups. Supplementary Figure S2: Bacterial composition, network analysis, and functional correlations between two groups. Supplementary Figure S3: Host association and differential abundance of viral clusters (VCs). Supplementary Figure S4: Volcano plot of differentially abundant metabolites in CF vs. ZF. Supplementary Table S1: Growth, feed efficiency and fatness traits in two groups. Supplementary Table S2: Comparative topological properties of the CF and ZF networks. Supplementary Table S3: Degree and eigenvector centrality of the hub nodes in CF and ZF networks. [file 40168_2026_2368_MOESM1_ESM.docx]

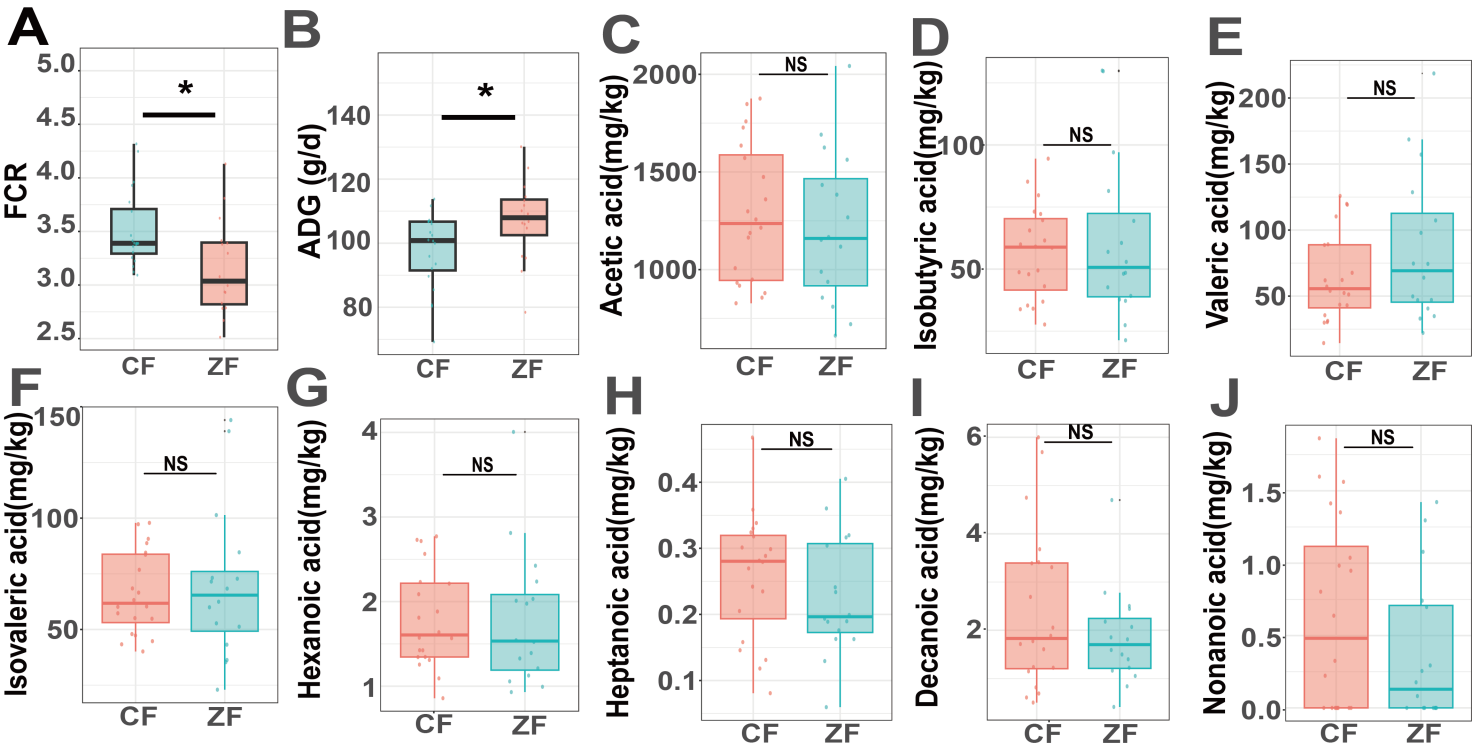


**Fig.S1** FE traits and SCFAs concentrations between two groups. **A** and **B.** Average daily gain (ADG) and feed conversion ratio (FCR) of CF (20) and ZF (16) ducks. **C** to **J.** Absolute concentrations (mg/kg) of eight SCFAs in cecal/intestinal contents: acetic acid, isobutyric acid, valeric acid, isovaleric acid, hexanoic acid, heptanoic acid, decanoic acid, and nonanoic acid. Statistical significance between groups is indicated (*p<0.05, **p<0.01, ***p<0.001).


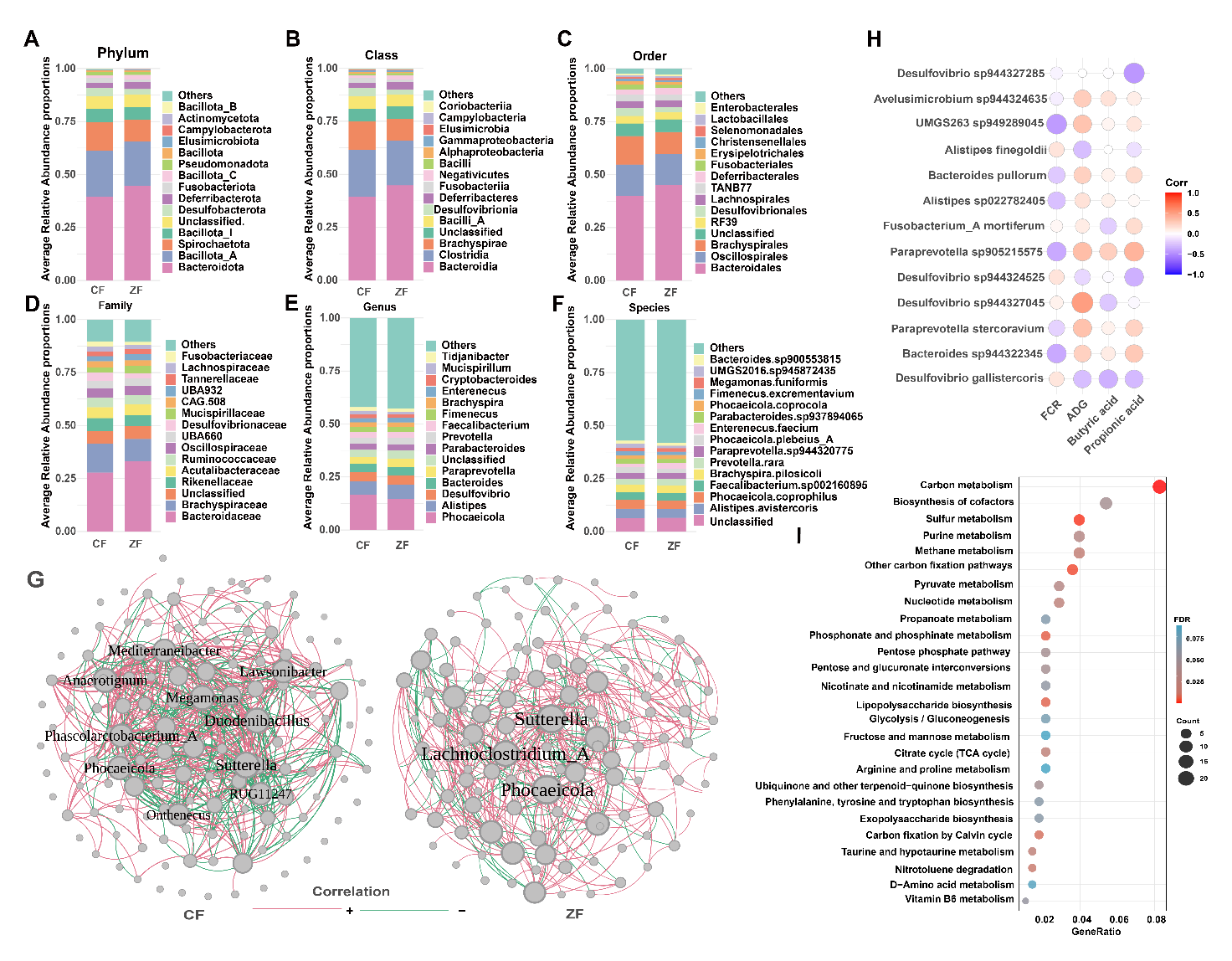


**Fig.S2** Bacterial composition, network analysis, and functional correlations between two groups. **A** to **F.** Relative abundance of the top 15 bacterial taxa at different taxonomic levels (Phylum, Class, Order, Family, Genus, and Species) based on metagenome sequencing. Each bar represents the average proportion within groups. **G.** Microbial co-occurrence networks, constructed using correlations (|r| ≥ 0.3, p < 0.05). Node size reflects degree centrality, and nodes with eigencentrality ≥ 0.8 are labeled. **H.** Correlations between differentially abundant bacteria and FE traits as well as SCFAs (butyrate and propionate). The color represents the correlation coefficient, which ranges from -1 to 1 with a gradient from blue to red. Deeper colors indicate stronger correlations. The size of the circle represents the p-value, with smaller p-values corresponding to larger circles. **I.** KEGG pathway enrichment analysis of differentially abundant KEGG orthologs (KOs).


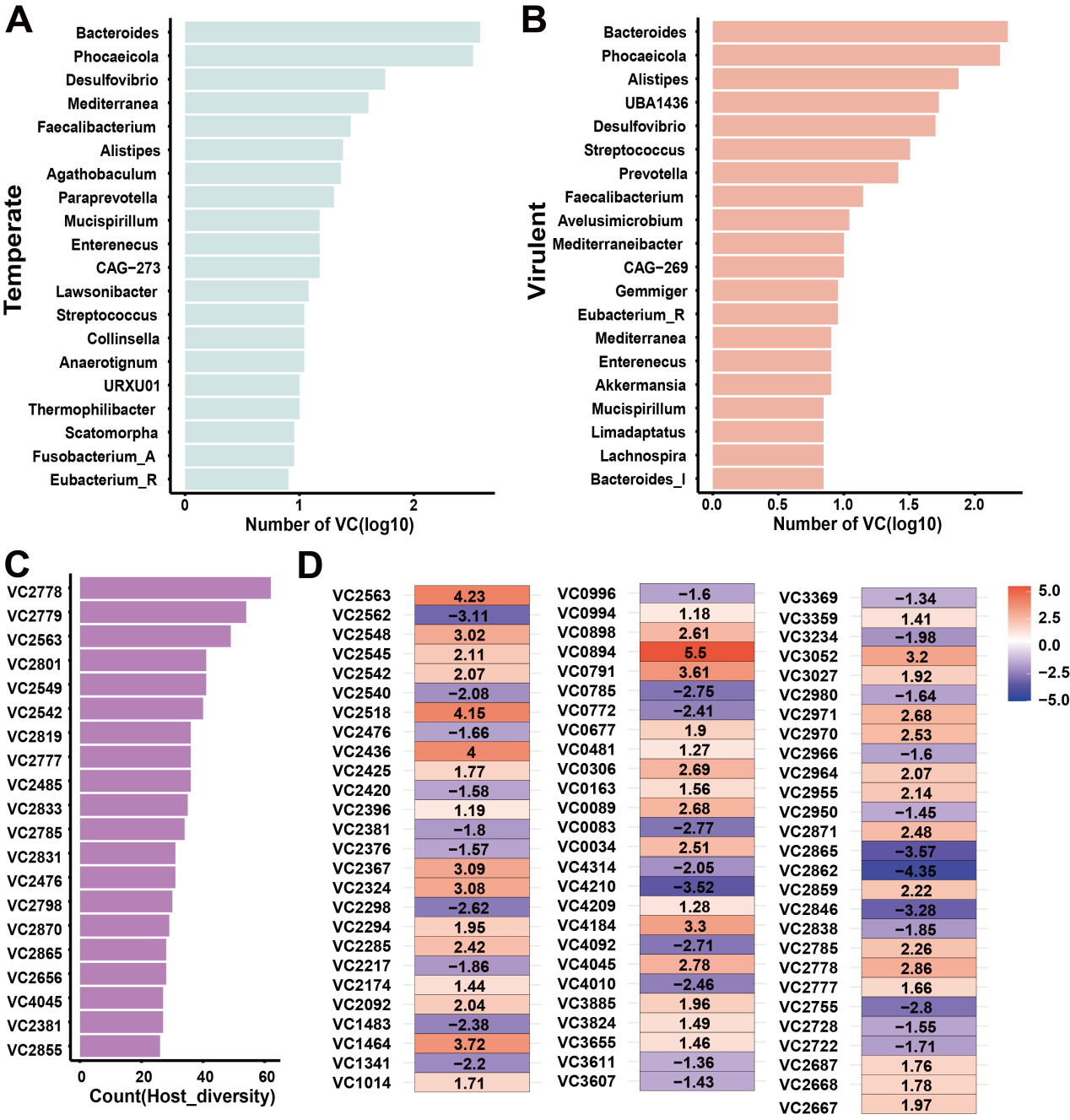


**Fig. S3** Host association and differential abundance of viral clusters (VCs). **A.** Number of temperate VCs with >50% completeness for the top 20 predicted hosts at the genus level. **B.** Number of virulent VCs with >50% completeness for the top 20 predicted hosts. **C.** Number of hosts associated with the top 20 VCs. **D.** Differentially abundant VCs between the two groups (adjust *p* < 0.05). Values represent natural log fold changes from ANCOM-BC analysis. The color gradient indicates the direction and magnitude of change: blue to white represents decreases, while white to red represents increases in ZF compared with CF.


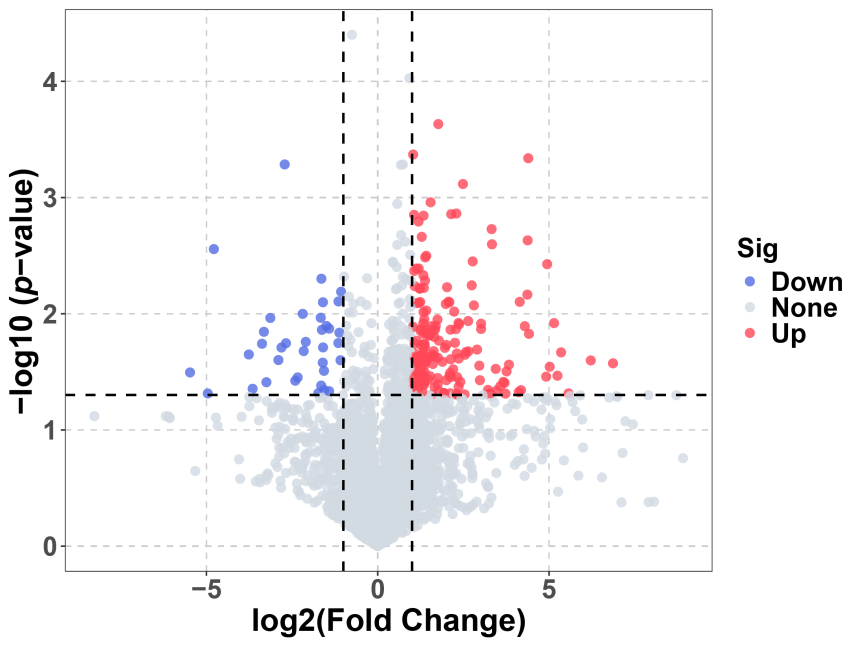


**Fig.S4** Volcano plot of differentially abundant metabolites in CF vs. ZF. Points above the dashed line represent metabolites with significant changes (|log_2_FC|≥1, *p* < 0.05). Metabolites are color-coded based on their expression status: blue for significantly downregulated metabolites, red for significantly upregulated metabolites, and gray for metabolites without significant changes.

**Table S1. Growth, feed efficiency and fatness traits in two groups**

|  | **CF** | |  | **ZF** | |  | **contrasts** | **RMSE** |
| --- | --- | --- | --- | --- | --- | --- | --- | --- |
|  | **LSM** | **SE** |  | **LSM** | **SE** |  | **p_value**  **(CF vs. ZF)** |  |
| Efficiency |  |  |  |  |  |  |  |  |
| ADFI, g/d | 348 | 1.96 |  | 330 | 2.07 |  | <0.0001 | 23.795 |
| ADG, g/d | 104 | 0.828 |  | 109 | 0.874 |  | <0.001 | 10.043 |
| FCR | 3.360 | 0.028 |  | 3.070 | 0.030 |  | <0.0001 | 0.339 |
| Fatness traits |  |  |  |  |  |  |  |  |
| AFW, g | 56.10 | 1.29 |  | 36.00 | 1.35 |  | <0.0001 | 605.791 |
| SFW, g | 713 | 8.13 |  | 563 | 8.53 |  | <0.0001 | 91.939 |
| AFP, % | 0.023 | <0.001 |  | 0.014 | <0.001 |  | <0.0001 | 0.006 |
| SFP, % | 0.289 | 0.002 |  | 0.222 | 0.002 |  | <0.0001 | 0.026 |

LSM least squares mean, SE standard error, RMSE root mean square error, ADFI average daily feed intake, ADG average daily gain, FCR average feed conversion ratio calculated as the ratio between ADFl and ADG, AFW abdominal fat weight, SFW subcutaneous fat weight, AFP abdominal fat percentage), SFP subcutaneous fat percentage.

**Table S2. Comparative Topological Properties of the CF and ZF Networks**

| **Comparative Topological Properties** | **CF** | **ZF** |
| --- | --- | --- |
| Number of nodes | 136 | 136 |
| Number of edges | 531 | 475 |
| Avg. number of neighbors | 10.019 | 8.523 |
| Network diameter | 7 | 6 |
| Network radius | 4 | 4 |
| Characteristic path length | 2.690 | 2.756 |
| Clustering coefficient | 0.443 | 0.336 |
| Network density | 0.095 | 0.077 |
| Network heterogeneity | 0.902 | 0.823 |
| Network centralization | 0.242 | 0.199 |

**Table S3 Degree and eigenvector centrality of the hub nodes in CF and ZF networks**

| **Nodes** | **Degree** | |  | **Eigenvector centrality** | |  |
| --- | --- | --- | --- | --- | --- | --- |
|  | **CF** | **ZF** |  | **CF** | **ZF** |  |
| **CF group** |  |  |  |  |  |  |
| *Sutterella* | 35 | 32 |  | 1 | 0.967 |  |
| *Duodenibacillus* | 34 | 16 |  | 0.952 | 0.459 |  |
| *Megamonas* | 32 | 25 |  | 0.922 | 0.780 |  |
| *Anaerotignum* | 32 | 11 |  | 0.914 | 0.367 |  |
| *Phocaeicola* | 29 | 30 |  | 0.868 | 1 |  |
| *Phascolarctobacterium_A* | 28 | 13 |  | 0.859 | 0.495 |  |
| *Mediterraneibacter* | 31 | 15 |  | 0.835 | 0.502 |  |
| *Lawsonibacter* | 27 | 15 |  | 0.827 | 0.459 |  |
| *Onthenecus* | 25 | 12 |  | 0.819 | 0.472 |  |
| *RUG11247* | 25 | 8 |  | 0.818 | 0.352 |  |
|  |  |  |  |  |  |  |
| **ZF group** |  |  |  |  |  |  |
| *Phocaeicola* | 29 | 30 |  | 0.868 | 1 |  |
| *Sutterella* | 35 | 32 |  | 1 | 0.966 |  |
| *Lachnoclostridium_A* | 4 | 23 |  | 0.102 | 0.859 |  |
